# Association between respiratory capacity, quality of life and cognitive function in elderly individuals

**DOI:** 10.31744/einstein_journal/2019AO4337

**Published:** 2019-01-28

**Authors:** Rayana de Oliveira Costa, Raphael Mendes Ritti-Dias, Gabriel Grizzo Cucato, Maysa Seabra Cendoroglo, Fabio Nasri, Maria Luiza Monteiro Costa, Luciana Diniz Nagem Janot de Matos, Fábio Gazelato de Mello Franco

**Affiliations:** 1 Universidade Federal do Vale do São Francisco, Petrolina, PE, Brazil.; 2 Universidade Nove de Julho, São Paulo, SP, Brazil.; 3 Hospital Israelita Albert Einstein, São Paulo, SP, Brazil.; 4 Universidade Federal de São Paulo, São Paulo, SP, Brazil.

**Keywords:** Aged, Quality of life, Cognition, Maximal voluntary ventilation, Idoso, Qualidade de vida, Cognição, Ventilação voluntária máxima

## Abstract

**Objective:**

To investigate associations between respiratory capacity, quality of life and cognitive function in elderly individuals.

**Methods:**

The sample included 386 elderly individuals (232 women). Respiratory capacity assessment was based on maximal expiratory pressure measured at peak expiratory flow. Subjects were classified according to peak expiratory flow values adjusted for sex, age and height of individuals with normal (peak expiratory flow curve <80% and >60%) or reduced (peak expiratory flow curve < 60%) respiratory capacity. The World Health Organization Quality of Life Questionnaire and the Mini-Mental State Examination were used to assess quality of life and cognitive function, respectively.

**Results:**

Elderly women with reduced respiratory capacity scored lower on the Mini-Mental State Examination (p=0.048) and quality of life questionnaire (p=0.040) compared to those with normal respiratory capacity. These differences were not observed in men (p>0.05).

**Conclusion:**

Reduced respiratory capacity was associated with poorer quality of life and cognitive function in elderly women. These associations were not observed in elderly men.

## RESUMO

**Objetivo:**

Investigar a associação entre capacidade respiratória, qualidade de vida e função cognitiva em idosos.

**Métodos:**

A amostra incluiu 386 idosos (232 mulheres). A capacidade respiratória foi avaliada pela pressão expiratória máxima obtida no pico de fluxo expiratório. Com base nos resultados máximos de fluxo expiratório, os indivíduos foram classificados de acordo com o valor previsto para sexo, idade e estatura em capacidade respiratória normal (curva de fluxo expiratório de pico inferior a 80% e superior a 60%) ou capacidade respiratória reduzida (curva de fluxo expiratório de pico menor que 60%). O questionário *World Health Organization Quality of Life* e o Miniexame do Estado Mental foram utilizados para avaliar a qualidade de vida e a função cognitiva, respectivamente.

**Resultados:**

Em comparação com mulheres idosas com capacidade respiratória normal, as pessoas com capacidade respiratória reduzida apresentaram escores mais baixos para o Miniexame do Estado Mental (p=0,048) e qualidade de vida (p=0,040). Nos homens, não foram observadas diferenças (p>0,05).

**Conclusão:**

Capacidade respiratória reduzida foi associada à pior qualidade de vida e à função cognitiva em mulheres idosas. Essas associações não foram observadas em idosos do sexo masculino.

## INTRODUCTION

Aging promotes lung structural and physiological changes associated with reduced lung function and respiratory capacity. Therefore, higher ventilatory demands imposed by small efforts often lead to dyspnea and fatigue in older adults. Also, respiratory impairment is the final stage of several conditions, such as pulmonary and cardiovascular diseases, cancer and many others.^(^ ^1^ ^,^ ^2^ ^)^

Clinical and population-based studies have shown that respiratory function is associated with cognitive disorders in older adults.^(^ ^3^ ^-^ ^5^ ^)^ These associations have been attributed to hypoxia-induced changes in the synthesis of neurotransmitters, such as acetylcholine, with resulting mental confusion and memory impairment.^(^ ^6^ ^)^ Reduced cognitive function have direct impacts on the quality of life of older adults.^(^ ^7^ ^,^ ^8^ ^)^ Hence, psycho-emotional disorders, such as low self-esteem and depression, may be associated with impaired respiratory function.

Evidence of reduced cognitive performance and poor quality of life in older adults with impaired respiratory function has been given.^(^ ^6^ ^)^ However, it is unclear whether these associations are sex-dependent. Elderly women have more comorbidities, faster respiratory function decline and higher prevalence of incapacity compared to elderly men.^(^ ^9^ ^)^ According to a recent study, peak expiratory flow (PEF) is associated with functional variables, such as handgrip strength and performance in the Timed Up and Go (TUG) test in women, but not in men.^(^ ^10^ ^)^

In this study, cognitive performance and quality of life of elderly men and women with preserved or reduced PEF were compared to investigate potential sex-related differences.

## OBJECTIVE

To investigate whether elderly women with decreased peak expiratory flow would have poorer cognitive function and quality of life compared to elderly men.

## METHODS

### Sample

The sample comprised 386 elderly individuals (232 women) recruited from a geriatric hospital in São Paulo (SP, Brazil). This hospital unit is specialized in care of institutionalized and community-dwelling adults aged over 60 years. A multidisciplinary team (physicians, physical therapists, psychologists, nurses, dietitians and kinesiology specialists) provides optimal health care and social support for elderly individuals.

The following inclusion criteria were adopted: age ≥65 years, absence of chronic obstructive pulmonary disease and absence of disability or signs of dementia based on clinical history or Mini-Mental State Examination (MMSE; cut-off point adjusted for level of education). Exclusion criteria were as follows: age <65 years, history of chronic obstructive pulmonary (bronchitis or emphysema), respiratory distress induced by moderate exercise, functional disability (bedridden or unable to move around the outpatient ward) and diagnosis of dementia based on medical team observations, clinical history or MMSE (cut-off point adjusted for level of education).

Procedures in this study were in compliance with the ethical standards of *Hospital Israelita Albert Einstein* Ethics Committee for Research Involving Human Experimentation and the Declaration of Helsinki (1975 version; 1983 revision), opinion no. 05/275, CAAE: 0051.0.028.000-05.

### Clinical and sociodemographic data

Clinical data ( *e.g* ., history of chronic diseases, current or past smoking habits) were extracted from medical history and physical examination records. Smoking was determined by multiplying the number of cigarette packs smoked per day by the number of years the individual has smoked. Individuals who had smoked at least 100 cigarettes in their lifetime but had quit smoking at the time of the interview were considered former smokers. Alcohol intake was determined according to units of alcohol (10mL) consumed per week; volumes of more than 14 units per week were considered excessive. Sociodemographic data (sex, age, education level, marital status and occupation) were also collected.

### Measurements

The level of depression was determined using the Brazilian version of the 15-item Geriatric Depression Scale (GDS short form)^(^ ^11^ ^)^ with a cut-off point of 3. Cognitive status assessment was based on the clock drawing test.^(^ ^12^ ^)^ Instrumental and basic activities of daily living were assessed using the Lawton-Brody and Katz questionnaires, respectively.^(^ ^13^ ^,^ ^14^ ^)^ The handgrip strength^(^ ^15^ ^)^ and the TUG tests^(^ ^16^ ^)^ were used to measure functional capacity.

### Expiratory flow

Peak expiratory flow measurements were obtained in the standing position. All subjects were instructed on the use of the peak flow meter (Assess^®^ Peak Flow Meter, Philips, Netherlands). Subjects were asked to breath-in deeply to full lung insufflation and, with the mouthpiece in place and the neck in neutral position ( *i.e* ., not flexed or extended), breathe out as hard as possible into the peak flow meter. The procedure was performed twice, and the highest value (L/min) was used in the analysis. The prediction equation of Neder et al.,^(^ ^17^ ^)^ was used to adjust upper and lower reference limits for age and sex. Peak expiratory flow is directly related to respiratory muscle strength; hence, PEF measurements are not diagnostic of obstructive or restrictive disease.

### Health-related quality of life

Health-related quality of life was assessed using the Brazilian version of the World Health Organization Quality of Life assessment (WHOQOL).^(^ ^18^ ^)^ This questionnaire comprises questions in six domains: physical health, psychological, level of independence, social relationships, environment and spirituality/religion/ personal beliefs. Domains are scored zero to 100 (worst and best possible quality of life, respectively). Participants answered the questionnaire themselves; assistance was limited to rereading questions slowly when required.

### Cognitive function

Cognitive function assessment was based on the MMSE. The MMSE comprises verbal (time-space orientation, immediate memory, evocation and procedural memory, attention and language) and nonverbal (perceptual-motor coordination and understanding instructions) responses. Scores range from zero to 30, with higher scores indicating better cognitive function. The cut-off point was determined according to level of educational, as described.^(^ ^19^ ^)^

### Statistical analysis

Data normality was investigated using the Shapiro-Wilk test. The following statistical procedures were used: mean, standard deviation, analysis of covariance (ANCOVA) for investigation of relations between quality of life, PEF and mental health, with adjustment for confounders, and the *t* -test for comparison between sexes. The frequency of chronic diseases and risk factors was analyzed using the χ ^2^ model. A level of significance of 0.05 (p<0.05) was adopted in inferential analyses. Statistical analyses were performed using the software Statistical Package for the Social Sciences (SPSS), version 20.

## RESULTS

Sample characteristics are shown in table 1 . Older adults with preserved or reduced expiratory flow had similar anthropometric and clinical parameters, except for higher prevalence of hypertension in men with reduced expiratory flow. Alcohol intake (p=0.013) and the incidence of stroke (p=0.023) were higher in women with reduced expiratory flow ( Table 2 ).

**Table 1:** Clinical characteristics of the sample

Characteristic	Men			Women		
	
	Preserved expiratory flow (n=86)	Reduced expiratory flow (n=69)	p value	Preserved expiratory flow (n=115)	Reduced expiratory flow (n=119)	p value
Age, years	73.6±7.6	79.1±8.1	0.051	74.2±8.2	79.0±8.9	0.070
Weight, kg	70.1±12.4	65.7±16.2	0.065	79.1±18.5	70.3±15.1	0.066
Height, m	1.54±0.7	1.53±0.6	0.097	1.65±0.9	1.57±0.1	0.119
Body mass index, kg/m^2^	29.4±4.9	28.1±6.3	0.053	29.2±5.9	28.9±5.2	0.106
Systolic BP, mmHg	144.9±26.3	146.6±25.4	0.133	147.5±24.0	142.6±24.9	0.165
Diastolic BP, mmHg	90.7±14.2	89.1±16.0	0.170	90.4±15.4	86.3±15.3	0.151
Heart rate, bpm/min	70.7±8.9	73.5±10.2	0.071	72.1±10.4	70.4±9.0	0.068
Smoking	33.8	29.3	0.606	52.3	41.1	0.107
Diabetes	25.0	19.7	0.450	25.0	17.7	0.199
Hypertension	57.5	71.1	<0.01	49.2	37.5	0.08
Coronary artery disease	5.0	3.9	1.00	18.9	11.5	0.144
Peripheral artery disease	2.5	5.3	0.434	4.5	3.1	0.737

Data are presented as percentil or mean±standard deviation. BP: blood pressure.

**Table 2:** Distribution of groups per age-related diseases

Age-related disease	Men (n=155)			Women (n=233)		
	
	Preserved expiratory flow (n=86)	Reduced expiratory flow (n=69)	p value	Preserved expiratory flow (n=115)	Reduced expiratory flow (n=119)	p value
Smoking	34.9	30.4	0.602	50.4	42.0	0.218
Alcohol abuse	20.9	18.8	0.781	41.7	26.1	0.013*
Stroke	0	0	0.100	2.6	3.4	0.023*
Ischemic stroke	3.5	2.9	0.837	0.9	2.5	0.736
Dementia	2.3	0	0.204	3.5	5.9	0.331
Depression	36.0	40.6	0.565	28.7	24.4	0.386
Diabetes	23.3	20.3	0.658	21.7	22.7	0.454
Coronary artery disease	5.8	2.9	0.187	20.0	12.6	0.128
Peripheral artery disease	2.3	4.3	0.480	5.2	2.5	0.126
Hypertension	55.8	29.0	0.001	45.2	42.9	0.285
Heart failure	7.0	5.8	0.167	9.6	6.7	0.227

* p<0.05. Data are present in frequency.

**Figure 1:**
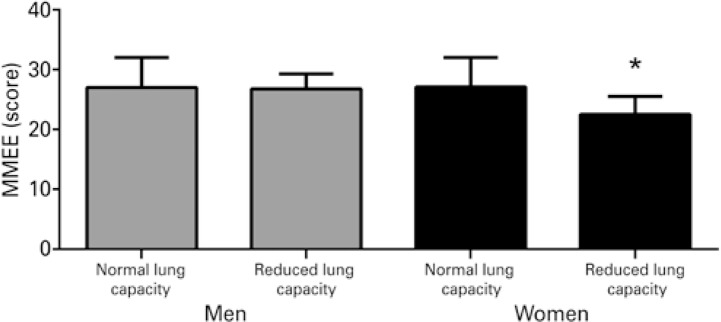
Mini Mental State Examination per expiratory flow

**Figure 2:**
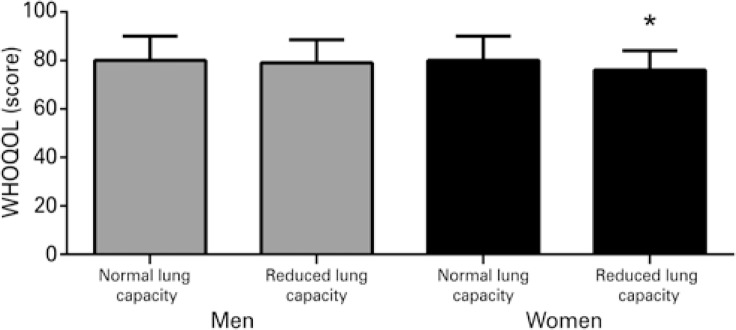
World Health Organization Quality of Life score per expiratory flow * p<0.05. WHOQOL: World Health Organization Quality of Life.

Comparisons of MMSE performance and health-related quality of life between elderly men and women with preserved or reduced expiratory flow are displayed in figures 1 and 2 . Elderly women with reduced expiratory flow performed worse on the MMSE (p=0.048) and had lower health-related quality of life scores (p=0.040). These differences were not observed in men (p>0.05).

* p<0.05. MMSE: Mini Mental State Examination.

## DISCUSSION

Poorer cognitive function and health-related quality of life in elderly women with reduced PEF compared to those with preserved expiratory flow was a major finding in this study. Interestingly, these differences were not observed in elderly men.

Cognitive impairment and physical dependence have been associated with adverse health outcomes, including poor quality of life and psychological disorders.^(^ ^20^ ^,^ ^21^ ^)^ Therefore, deeper understanding of factors potentially associated with cognitive and physical decline may contribute to the development of effective practices and therapies, aiming to improve health-related quality of life and promote functional independence in older adults. In this study, women with reduced PEF had poorer cognitive function. In a study of Yaffe et al.,^(^ ^22^ ^)^ respiratory disorders were a strong predictor of mild cognitive impairment and dementia among 298 women with respiratory disorders. These findings may reflect progressive respiratory strength and skeletal muscle mass loss in women, particularly after menopause, leading to hypoxia-induced cognitive deficits.^(^ ^23^ ^-^ ^25^ ^)^ A potential underlying mechanism is the rapid decrease in circulating levels of hormones, such as testosterone, in women aged 20 to 45 years.^(^ ^26^ ^,^ ^27^ ^)^ Testosterone, a major anabolic hormone, plays a significant role in protein synthesis in skeletal muscle and in muscle regeneration.^(^ ^28^ ^)^

Mechanisms underpinning the effects of hypoxemia on cognitive function in women are difficult to address in samples such as the one in this study, due to age-related comorbidities, as obesity, *diabetes mellitus* , hypertension and frailty.^(^ ^29^ ^)^ Nonetheless, changes in the prefrontal cortex may be involved, as suggested by Beebe et al.,^(^ ^30^ ^)^ Oxidative stress is thought to promote homeostatic imbalances and neuronal changes in specific areas, particularly the prefrontal cortex, with resulting dysregulation of the executive system and decline in primary cognitive abilities.^(^ ^29^ ^)^

Also, health-related quality of life was poorer in elderly women with reduced PEF compared to those with preserved PEF in this sample. The relation between health-related of quality of life and respiratory function was investigated by Renwick et al.^(^ ^31^ ^)^ In that study, women with preserved respiratory function had higher health-related quality of life scores, possibly due to associations between health-related of quality of life and physical capacity, given the negative impacts of dyspnea and fatigue resulting from respiratory muscle weakness on performance of daily activities.

Interestingly enough, cognitive function and health-related quality of life did not differ between men with preserved or reduced PEF. Reasons behind such sex-related differences remain to be determined. Faster muscle mass loss has been reported in postmenopausal women.^(^ ^32^ ^)^ Also, women have higher numbers of frailty markers and higher prevalence of frailty compared to men.^(^ ^33^ ^)^ Aforementioned factors may enhance negative effects of reduced respiratory strength on expiratory flow.

This study has some limitations. First, its cross-sectional design precluded inferences of causality. Second, the sample involved different risk factors, and data regarding use of medication were lacking, precluding further analysis of these variables. Finally, PEF is a measure of respiratory strength and does not allow lung volume and capacity determination.

## CONCLUSION

Elderly women with reduced peak expiratory flow had poorer health-related quality of life and cognitive function compared with those with preserved expiratory flow. Similar differences were not observed in elderly men.

Further studies are warranted to clarify these findings, including more accurate assessment of potential interfering variables, such as medication use and lifestyle habits.

## References

[1] Taffet GE, Donohue JF, Altman PR (2014). Considerations for managing chronic obstructive pulmonary disease in the elderly. Clin Interv Aging.

[2] Gorzoni ML, Freitas EV, Py L (2016). O envelhecimento respiratório. Tratado de Geriatria e Gerontologia.

[3] Cleutjens FA, Ponds RW, Spruit MA, Burgmans S, Jacobs HI, Gronenschild EH (2017). The relationship between cerebral small vessel disease, hippocampal volume and cognitive functioning in patients with COPD: An MRI study. Front Aging Neurosci.

[4] Cleutjens FA, Wouters EF, Dijkstra JB, Spruit MA, Franssen FM, Vanfleteren LE (2014). The COgnitive-Pulmonary Disease (COgnitive-PD) study: protocol of a longitudinal observational comparative study on neuropsychological functioning of patients with COPD. BMJ Open.

[5] Zhang X, Cai X, Shi X, Zheng Z, Zhang A, Guo J (2016). Chronic obstructive pulmonary disease as a risk factor for cognitive dysfunction: a meta-analysis of current studies. J Alzheimers Dis.

[6] Torres-Sánchez I, Rodríguez-Alzueta E, Cabrera-Martos I, López-Torres I, Moreno-Ramírez MP, Valenza MC (2015). Cognitive impairment in COPD: a systematic review. J Bras Pneumol.

[7] Pereira RJ, Cotta RM, Franceschini SC, Ribeiro RC, Sampaio RF, Priore SE (2006). Contribuição dos domínios físico, social, psicológico e ambiental para a qualidade de vida global de idosos. Rev Psiquiatr.

[8] Carneiro RS, Falcone E, Clark C, Del Prette Z, Del Prette A (2007). Qualidade de vida, apoio social e depressão em idosos: relação com habilidades sociais. Psicol Reflex Crit.

[9] Santos DC, Bianchi LR (2014). Envelhecimento morfofuncional: diferença entre os gêneros. Arq MUDI.

[10] Ritti-Dias RM, Cucato GG, de Mello Franco FG, Cendoroglo MS, Nasri F, Monteiro-Costa ML (2016). Peak expiratory flow mediates the relationship between handgrip strength and timed up and go performance in elderly women, but not men. Clinics (São Paulo).

[11] Yesavage JA, Brink TL, Rose TL, Lum O, Huang V, Adey M Development and validation of a geriatric depression screening scale: a preliminary report. J Psychiatr Res.

[12] Manos PJ, Wu R (1994). The ten point clock test: a quick screen and grading method for cognitive impairment in medical and surgical patients. Int J Psychiatry Med.

[13] Lawton MP, Brody EM (1969). Assessment of older people: self-maintaining and instrumental activities of daily living. Gerontologist.

[14] Katz S, Downs TD, Cash HR, Grotz RC (1970). Progress in development of the index of ADL. Gerontologist.

[15] Rantanen T, Era P, Heikkinen E (1994). Maximal isometric strength and mobility among 75-year-old men and women. Age Ageing.

[16] Podsiadlo D, Richardson S (1991). The timed “Up & Go”: a test of basic functional mobility for frail elderly persons. J Am Geriatr Soc.

[17] Neder JA, Andreoni S, Lerario MC, Nery LE (1999). Reference values for lung function tests. II. Maximal respiratory pressures and voluntary ventilation. Braz J Med Biol Res.

[18] The Whoqol Group (1998). The World Health Organization Quality of Life assessment (WHOQOL): development and general psychometric properties. Soc Sci Med.

[19] Brucki SM, Nitrini R, Caramelli P, Bertolucci PH, Okamoto IH (2003). Suggestions for utilization of the mini-mental state examination in Brazil. Arq Neuropsiquiatr.

[20] Gorska-Ciebiada M, Saryusz-Wolska M, Ciebiada M, Loba J (2014). Mild cognitive impairment and depressive symptoms in elderly patients with diabetes: prevalence, risk factors, and comorbidity. J Diabetes Res.

[21] Langlois F, Vu TT, Kergoat MJ, Chassé K, Dupuis G, Bherer L (2012). The multiple dimensions of frailty: physical capacity, cognition, and quality of life. Int Psychogeriatr.

[22] Yaffe K, Laffan AM, Harrison SL, Redline S, Spira AP, Ensrud KE (2011). Sleep-disordered breathing, hypoxia, and risk of mild cognitive impairment and dementia in older women. JAMA.

[23] Albuquerque IM, Emmanouilidis A, Ortolan T, Cardoso DM, Gass R, Jost RT (2013). Capacidade funcional submáxima e força muscular respiratória entre idosas praticantes de hidroginástica e dança: um estudo comparativo. Rev Bras Geriatr Gerontol.

[24] Yaffe K, Sawaya G, Lieberburg I, Grady D (1998). Estrogen therapy in postmenopausal women: effects on cognitive function and dementia. JAMA.

[25] Viegas CA (2010). Epidemiologia dos distúrbios respiratórios do sono. J Bras Pneumol.

[26] Vitale G, Cesari M, Mari D (2016). Aging of the endocrine system and its potential impact on sarcopenia. Eur J Intern Med.

[27] Morley JE, Perry HM (2003). Androgens and women at the menopause and beyond. J Gerontol A Biol Sci Med Sci.

[28] La Colla A, Pronsato L, Milanesi L, Vasconsuelo A (2015). 17 β-Estradioland testosterone in sarcopenia: role of the satellite cells. Ageing Res Rev.

[29] Fried LP, Tangen CM, Walston J, Newman AB, Hirsch C, Gottdiener J, Seeman T, Tracy R, Kop WJ, Burke G, McBurnie MA, Cardiovascular Health Study Collaborative Research Group (2001). Frailty in older adults: evidence for a phenotype. J Gerontol A Biol Sci Med Sci.

[30] Beebe DW, Gozal D (2002). Obstructive sleep apnea and the prefrontal cortex: towards a comprehensive model linking nocturnal upper airway obstruction to daytime cognitive and behavioral deficits. J Sleep Res.

[31] Renwick DS, Connolly MJ (1996). Impact of obstructive airways disease on quality of life in older adults. Thorax.

[32] Chen HI, Kuo CS (1989). Relationship between respiratory muscle function and age, sex, and other factors. J Appl Physiol (1985).

[33] Langholz PL, Strand BH, Cook S, Hopstock LA (2018). Frailty phenotype and its association with all-cause mortality in community-dwelling Norwegian women and men 70 years and older: the Tromsø study 2001-2016. Geriatr Gerontol Int.

